# Differentiating triple-negative breast cancer and atypical fibroadenomas using an ultrasound-based radiomics nomogram

**DOI:** 10.1186/s12880-025-01963-z

**Published:** 2025-10-14

**Authors:** Xinyu Zhu, Kai Zhu, Wenjia Wan, Zhicheng Ran, Dongmo Wang

**Affiliations:** 1 Ultrasound Department, Second Affiliated Hospital of Harbin Medical University, No 246 Xue Fu Road, Nan Gang District, Harbin, 150086 Heilongjiang Province China; 2 Radiology Department, First Affiliated Hospital of Harbin Medical University, No 23 You Zheng Street, Nan Gang District, Harbin, 150001 Heilongjiang Province China

**Keywords:** Atypical fibroadenomas, Nomogram, Radiomics, Triple-negative breast cancer, Ultrasound

## Abstract

**Objective:**

To investigate the application value of ultrasound radiomics technology in differentiating TNBC from atypical fibroadenoma (AFA) and to develop a nomogram model.

**Methods:**

In this study, 75 patients with TNBC and 90 patients with AFA who underwent surgery were enrolled and randomly divided into training (*n* = 114) and validation cohorts (*n* = 51). Radiomic features were extracted from the images. LASSO regression analysis and stepwise regression were used for features selection. A prediction model was developed by combining multivariate logistic regression with the selected imaging biomarkers, resulting in the generation of a nomogram. A confusion matrix was used to visualize the distribution of correct and misclassified classifications. Finally, the validity of the model was assessed by using the receiver operator characteristic curve and calibration curve.

**Results:**

Multivariate analysis based on ultrasound features identified elasticity score, fibrous strands within the mass and lateral acoustic shadow as independent factors for differentiating TNBC from AFA. The radiomics signature, composed of 4 selected features, achieved good diagnostic performance. The nomogram incorporating the radiomics signature demonstrated favorable diagnostic efficacy, and the AUC of the training set and the validation set were 0.977 and 0.982, which outperformed the ultrasound model (AUC = 0.939). The calibration curve demonstrated the good clinical utility of the radiomics nomogram.

**Conclusion:**

This study systematically analysed the ability of ultrasound radiomics to differentiate TNBC from AFA and the ultrasound-based radiomics nomogram effectively improved the diagnostic accuracy of TNBC.

## Introduction

Breast cancer is the most common malignancy in women worldwide, with its incidence and mortality steadily increasing over the past few decades. According to the most recent survey data from the International Agency for Research on Cancer (IARC, 2022), nearly 20 million new cancer cases were diagnosed globally, among which breast cancer accounted for more than 2.3 million new cases and approximately 660,000 deaths annually, posing a substantial threat to women’s physical and psychological well-being [1]. Early detection, accurate diagnosis, and timely treatment are therefore essential to improve outcomes and survival.

Triple-negative breast cancer (TNBC) is a distinct molecular subtype of breast cancer that represents approximately 15–20% of all cases [2]. Characterized by the absence of estrogen receptor (ER), progesterone receptor (PR), and human epidermal growth factor receptor 2 (HER2), TNBC exhibits highly aggressive biological behavior, a high recurrence rate, and poor prognosis. Early and accurate diagnosis is thus critical for patient management. However, TNBC shares several overlapping ultrasound features with atypical fibroadenomas (AFAs), such as irregular shape, non-parallel orientation, and heterogeneous internal structure [3, 4]. These similarities frequently result in diagnostic challenges and misclassification. Consequently, comprehensive assessment combining ultrasound with additional imaging modalities or histopathological evaluation is often required to enhance diagnostic accuracy in clinical practice [5].

Radiomics, initially developed in computed tomography (CT) and magnetic resonance imaging (MRI), has demonstrated significant potential in extracting high-dimensional quantitative features that reflect tumor heterogeneity and biological behavior. With the rapid advancement of machine learning and artificial intelligence, radiomics has been increasingly applied in oncology [6].Ultrasound radiomics, an emerging computational imaging technology, enables the extraction of numerous quantitative features from medical images for disease diagnosis and prognostic prediction [7]. Compared with conventional ultrasound interpretation, radiomics offers richer, more comprehensive information, thereby reducing observer dependence and minimizing diagnostic subjectivity [3]. Beyond morphological assessment, radiomics features may also capture information related to tumor molecular profiles and functional characteristics, providing clinicians with a broader and more reliable diagnostic basis [8]. This approach improves diagnostic consistency and objectivity, aids in personalised treatment planning, and enhances prognostic evaluation, ultimately contributing to improved patient outcomes. Currently, ultrasound radiomics has been applied in several malignancies, including lung, prostate, and brain cancers, with promising results [9, 10].

In light of these advances, ultrasound radiomics shows great promise in distinguishing TNBC from AFA. However, research addressing this application specifically is limited. Accurate differentiation between these two conditions is essential for clinical practice, as it influences treatment decisions, prevents unnecessary invasive procedures and reduces the risk of misdiagnosis. Therefore, the aim of this study was to investigate the diagnostic value of ultrasound radiomics in distinguishing TNBC from AFA and to establish a radiomics-based diagnostic model. By addressing this clinically relevant challenge, the study seeks to provide clinicians with a more accurate and objective diagnostic tool to support clinical decision-making and optimise patient management [11, 12].

## Materials and methods

### Patients

#### Sample selection and sample size

**Fig. 1:**
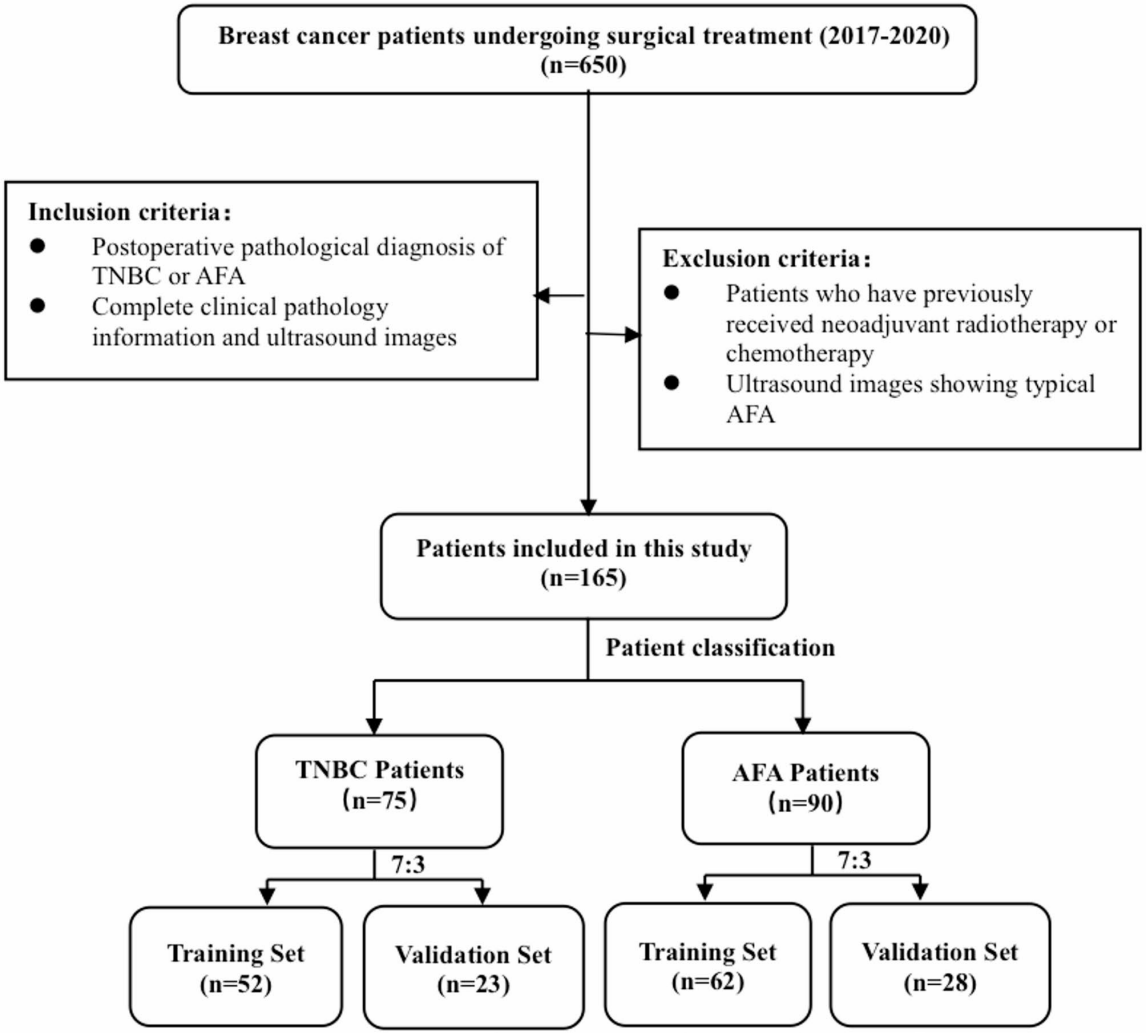
Flow chart of case screening

A total of 650 patients with breast tumours who underwent surgery at the Second Affiliated Hospital of Harbin Medical University between January 2017 and January 2020 were reviewed. Based on the study objectives and exclusion criteria, 165 patients were ultimately enrolled and randomly divided into training and validation cohorts at a ratio of 7:3. Of these patients, 75 were diagnosed with TNBC (training cohort: 52; validation cohort: 23) and 90 with AFA (training cohort: 62; validation cohort: 28). The patient selection process is illustrated in Fig. 1.

#### Eligibility criteria

Patients were eligible if they met the following criteria: (1) postoperative pathological confirmation of TNBC or fibroadenoma; and (2) availability of complete clinicopathological data and ultrasound images. Exclusion criteria included: (1) prior neoadjuvant radiotherapy or chemotherapy; and (2) ultrasound images demonstrating typical fibroadenoma characteristics, namely an oval or round shape with a smooth, well-defined tumor margin [13, 14].

#### Ultrasonic features

All patients underwent ultrasound examination using the Hitachi Vision 900 system (Hitachi Medical Systems, Tokyo, Japan) with a 5–12 MHz linear high-frequency probe. According to the BI-RADS classification, ultrasound features of the tumor were assessed, including shape, orientation, tumor margin, lesion boundary, echo pattern, posterior acoustic features, architectural distortion of the surrounding tissue, and calcification. Two sonographers, each with more than 5 years of experience in breast ultrasound diagnosis, independently reviewed the ultrasound images. Both were blinded to patients’ clinical data and pathological immunohistochemistry results, and consensus was reached through discussion.

In addition, three clinically valuable features were evaluated based on prior reports for distinguishing fibroadenomas from malignant breast tumors: circumferential blood flow, fibrous strands, and lateral shadow [15]. Circumferential blood flow was defined as the presence of blood vessels within 0.5 cm around the tumor; more than two visible vessels were defined as positive, whereas fewer than two or absent blood flow was defined as negative [16, 17]. Fibrous strands referred to linear or cord-like fibrous tissue within nodules, which is particularly valuable due to the high fibrous content in fibroadenomas [18, 19]. Elastography was also applied, and scoring was performed using a 5-point scale. For this study, elastography scores were dichotomized as < 3 and ≥ 3 [20].Furthermore, color Doppler flow imaging (CDFI) was graded according to Adler’s blood flow criteria, with blood flow distribution categorized as sparse (Adler 0–I) or abundant (Adler II–III) [21].

#### Extracting ultrasound radiomics features

①Region of Interest (ROI) delineation: first, pre-process the ultrasound images, including denoising and grey scale standardisation, to ensure consistency and accuracy of feature extraction; then, import the DICOM images into 3D Slicer 5.2.1 software and select the image that shows the largest diameter of the mass and is clear for delineation of the ROI [22].

②Feature extraction: Radiomic features of ROIs were extracted from ultrasound images using the PyRadiomics package within the 3D Slicer software environment. The ultrasound radiomics features included First Order Statistics, 2D Shape-based, gray-level cooccurrence matrix (GLCM), Gray-level size-zone matrix (GLSZM), gray-level run-length matrix (GLRLM), and gray-level dependence matrix (GLDM), gray tone difference matrix(GTDM) and so on [23, 24]. Finally, 860 variables were extracted for each patient.

③Feature selection: To mitigate dimensionality and overfitting, feature selection was performed. First, Least Absolute Shrinkage Selection Operator (LASSO) regression with 10-fold cross-validation was applied to the entire feature set in the training cohort. The optimal regularization parameter (lambda) was chosen based on the minimum mean squared error criterion. This process retained features with non-zero coefficients. Subsequently, the LASSO-selected features underwent stepwise backward selection within a multivariate logistic regression framework (retention threshold: *P* < 0.05). Ultimately, four key radiomic features were identified for model building [25].

#### Model development and evaluation

Ultrasound-based model: Univariate logistic regression was applied to ultrasound features in the training cohort, and variables with *P* < 0.05 were included in multivariable logistic regression. Independent predictors were identified using backward stepwise selection with the minimum Akaike information criterion (AIC) [26]. The final model was evaluated in both training and validation sets by receiver operating curve (ROC) analysis [area under the curve (AUC) with 95% confidence intervals (CIs)] and calibration assessment using calibration curves and the Hosmer-Lemeshow goodness-of-fit test (*P* >0.05 indicating adequate fit) [27, 28].

**Fig. 2:**
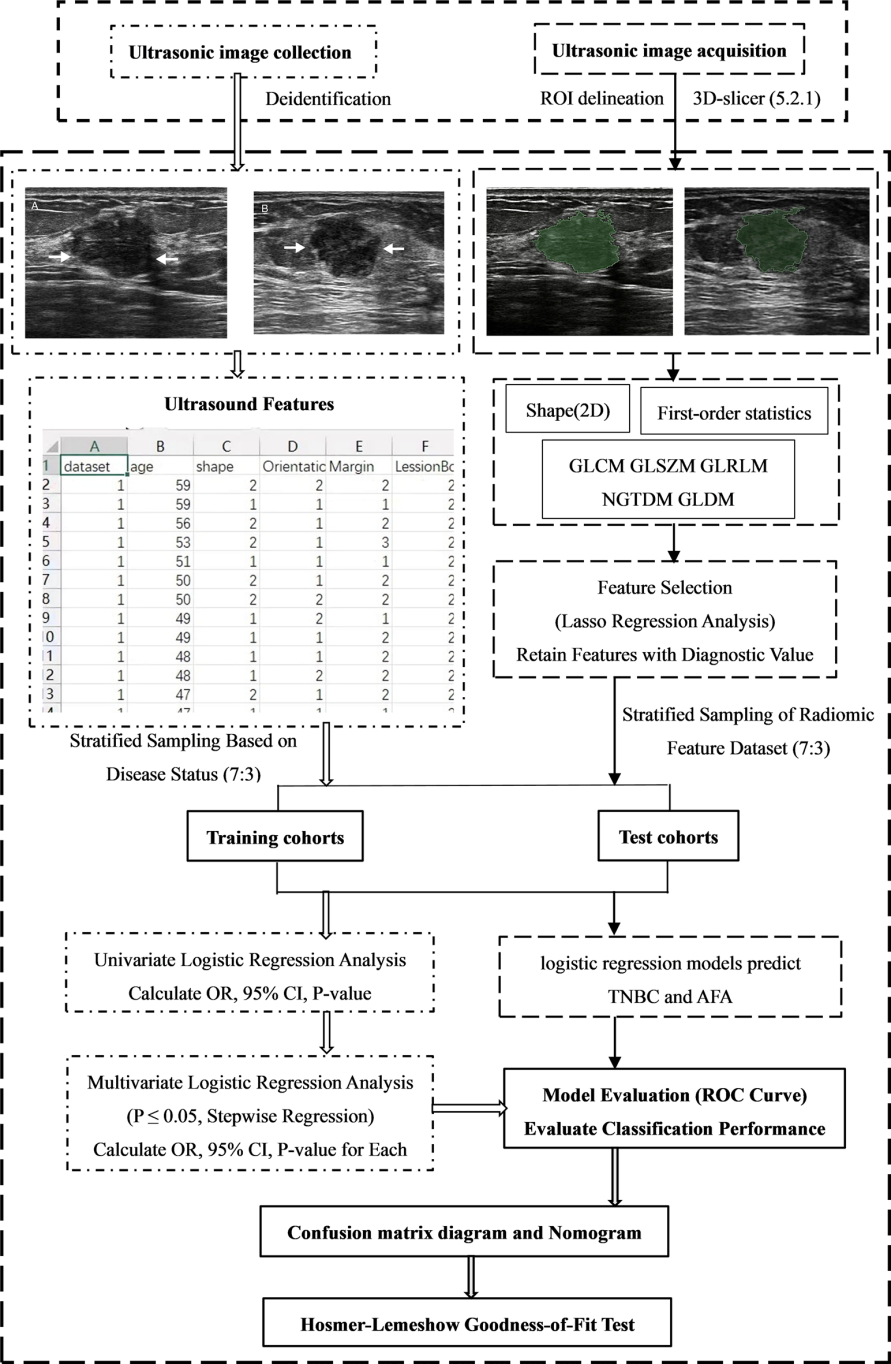
Features extraction screening and models development flowchart

Radiomics signature model: Radiomic features were divided into training and validation cohorts. Feature selection was performed using LASSO regression implemented in the glmnet package. The optimal penalty parameter (λ) was selected by ten-fold cross-validation (cv.glmnet function), with both λ.min (minimum error) and λ.1se (within one standard error of the minimum) considered. Cross-validation was stratified by outcome to preserve class balance, and a fixed random seed (521) was applied to ensure reproducibility. Dimensionality reduction was performed on the training set using the LASSO regression method with the optimal penalty parameter (λ). Non-zero features were then refined using stepwise regression to produce the final subset. A nomogram incorporating the selected features was constructed, and its performance was evaluated by AUC, calibration curves, and the Hosmer–Lemeshow test [29]. The workflow of feature extraction and model construction is illustrated in Fig. 2.

### Statistical analysis

Statistical analyses were conducted using R software (version 4.3.2). Continuous variables were presented as mean ± standard deviation, and categorical variables as counts and percentages. Independent samples t-tests and chi-squared tests were used to compare continuous and categorical variables, respectively. Radiomic features were initially reduced via least absolute shrinkage selection operator (LASSO) regression, followed by stepwise selection. A multivariable logistic regression model was then constructed, and ROC curves were generated for the training and validation cohorts. Model performance was assessed using the area under the curve (AUC), sensitivity, specificity, positive predictive value (PPV), negative predictive value (NPV), and overall accuracy. Calibration was assessed using calibration curves and the Hosmer-Lemeshow goodness-of-fit test. Differences were considered statistically significant at *P*<0.05.

## Results

### Patient characteristics

A total of 165 patients, including 90 with atypical fibroadenoma (AFA) and 75 with triple-negative breast cancer (TNBC), were enrolled. They were randomly assigned to a training cohort (*n* = 114; AFA: 62, TNBC: 52) and a validation cohort (*n* = 51; AFA: 28, TNBC: 23) at a 7:3 ratio. The median age was 51 years (range 16–87) in the training set and 46 years (range 17–75) in the validation set, with no significant difference observed (*P* > 0.05).

### Ultrasound features

**Fig. 3:**
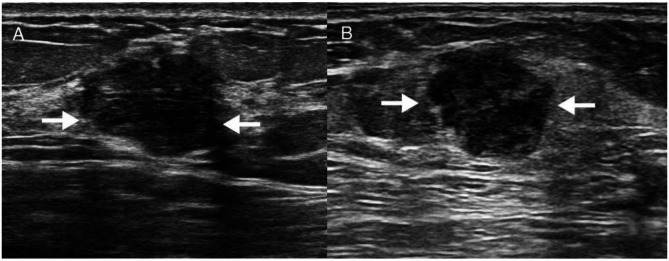
(**A**) Ultrasound images of patients. AFA with irregular morphology, posterior echo-enhancement and lateral shadow (white arrows); (**B**) TNBC with irregular morphology, posterior echo-enhancement and no lateral shadow (white arrows)

**Fig. 4:**
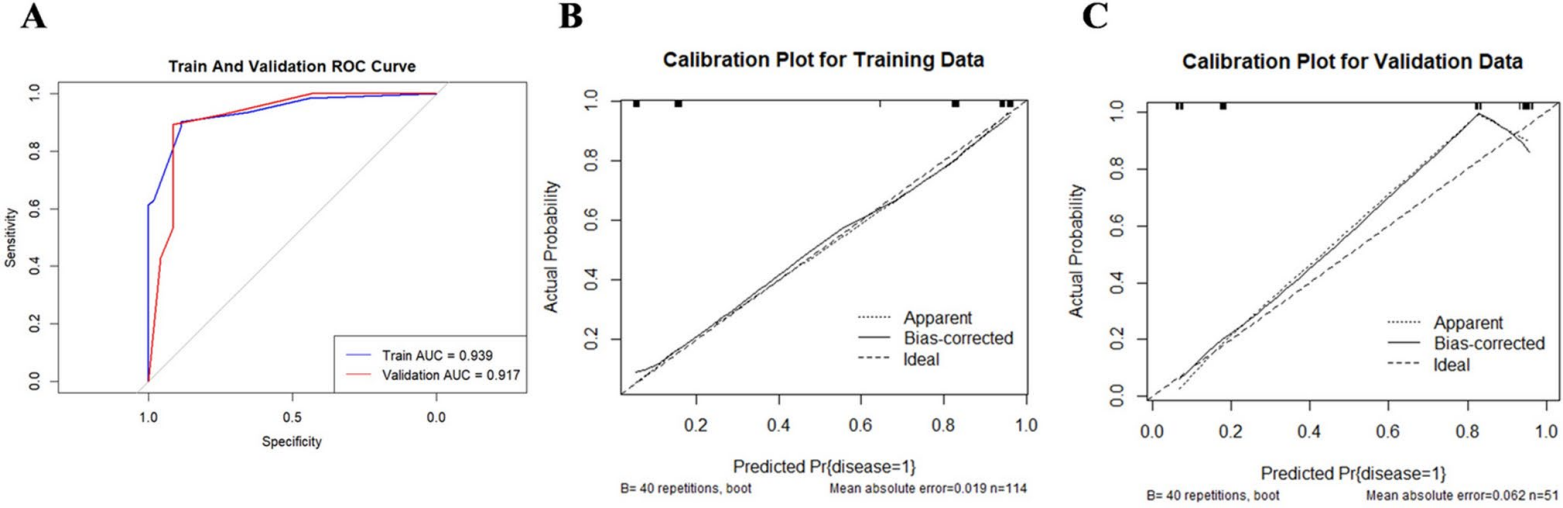
(**A**) ROC curves of the US-based model in the training cohort (blue curve) and validation cohort (red curve); (**B**, **C**) The calibration curves of the US-based model in the training cohort and validation cohort

The univariate analysis of ultrasound features for TNBC and AFA is presented in Table 1. Ten features showed statistically significant differences between the two groups (*P* < 0.05): shape, tumor margin, echo pattern, posterior acoustic features, calcification, blood flow grade, elasticity score, circumferential blood flow, fibrous strands, and lateral shadow. Based on these results, multivariable analysis was conducted, identifying elasticity score (OR 0.02; 95% CI 0.00–0.29; *P* = 0.003), fibrous strands (OR 0.11; 95% CI 0.02–0.53; *P* = 0.006), and lateral shadow (OR 0.18; 95% CI 0.04–0.78; *P* = 0.022) as independent predictors for distinguishing TNBC from AFA (Table 2; Fig. 3).Based on the multivariable regression results from the training cohort, a diagnostic model was developed, and its performance was assessed using ROC curves. The area under the curve (AUC) was 0.939 (95% CI 0.90–0.98) for the training set and 0.917 (95% CI 0.83–0.99) for the validation set (Fig. 4A). Calibration curves showed a strong correlation between the predicted and observed outcomes (Hosmer-Lemeshow test: *P* = 0.440; Fig. 4B and C).

**Table 1:** Univariate analysis results of ultrasound features for TNBC and AFA

Variables *n* (%)	Training cohort (*n* = 114)			Validation cohort (*n* = 51)			*P*
	TNBC (*n* = 52)	AFA (*n* = 62)	*P*	TNBC (*n* = 23)	AFA (*n* = 28)	*P*	
**Shape**			< 0.001			0.244	< 0.001
Regular	4 (7.69)	34 (54.84)		7 (30.43)	13 (46.43)		
Irregular	48 (92.31)	28 (45.16)		16 (69.57)	15 (53.57)		
**Orientation**			0.557			0.082	0.113
Parallel	36 (69.23)	46 (74.19)		15 (65.22)	25 (89.29)		
Nonparallel	16 (30.77)	16 (25.81)		8 (34.78)	3 (10.71)		
**Tumor margin**			< 0.001			< 0.001	< 0.001
Smooth	0 (0.00)	27 (43.55)		0 (0.00)	14 (50.00)		
Microlobulated	42 (80.77)	33 (53.23)		21 (91.30)	14 (50.00)		
Angular/speculated	10 (19.23)	2 (3.22)		2 (8.70)	0 (0.00)		
**Lesion boundary**			0.298			1.000	0.192
Echogenic rim	1 (1.92)	5 (8.06)		0 (0.00)	1 (3.57)		
abrupt interface	51 (98.08)	57 (91.94)		23 (100.00)	27 (96.43)		
**Echo pattern**			0.129			0.010	0.007
Hypoechoic	24 (46.15)	20 (32.26)		14 (60.87)	7 (25.00)		
Other echo	28 (53.85)	42 (67.74)		9 (39.13)	21 (75.00)		
**Posterior Acoustic Features**			< 0.001			0.001	< 0.001
Shadowing	29 (55.77)	10 (16.13)		13 (56.52)	4 (14.29)		
Enhancement	23 (44.23)	51 (82.26)		10 (43.48)	24 (85.71)		
No change	0 (0.00)	1 (1.61)		0 (0.00)	0 (0.00)		
**Architectural distortion of the surrounding tissue**			0.810			0.495	0.950
Yes	4 (7.69)	3 (4.84)		0 (0.00)	2 (7.14)		
No	48 (92.31)	59 (95.16)		23 (100.00)	26 (92.86)		
**Calcification**			< 0.001			1.000	0.003
Preserved	23 (44.23)	10 (16.13)		5 (21.74)	5 (17.86)		
Absent	29 (55.77)	52 (83.87)		18 (78.26)	23 (82.14)		
**Blood flow grade**			< 0.001			0.741	< 0.001
Adler 0 ~ I	42 (80.77)	29 (46.77)		15 (65.22)	17 (60.71)		
Adler II ~ III	10 (19.23)	33 (53.23)		8 (34.78)	11 (39.29)		
**Elasticity score**			< 0.001			< 0.001	< 0.001
< 3	1 (1.92)	40 (64.52)		2 (8.70)	18 (64.29)		
≥ 3	51 (98.08)	22 (35.48)		21 (91.30)	10 (35.71)		
**Circumferential blood flow**			0.013			0.137	0.004
Yes	26 (50.00)	17 (27.42)		13 (56.52)	10 (35.71)		
No	26 (50.00)	45 (72.58)		10 (43.48)	18 (64.29)		
**Fibrous strands**			< 0.001			< 0.001	< 0.001
Yes	17 (32.69)	56 (90.32)		6 (26.09)	23 (82.14)		
No	35 (67.31)	6 (9.68)		17 (73.91)	5 (17.86)		
**Lateral shadow**			< 0.001			< 0.001	< 0.001
Yes	17 (32.69)	55 (88.71)		8 (34.78)	24 (85.71)		
No	35 (67.31)	7 (11.29)		15 (65.22)	4 (14.29)		

**Table 2:** Multivariate logistic regression analysis results for the training set

Variables	β	S. E	Z	*P*	OR (95% CI)
Elastic	-3.75	1.28	-2.94	0.003	0.02 (0.00 -0.29)
Fibrous strands	-2.23	0.81	-2.75	0.006	0.11 (0.02–0.53)
lateral shadow	-1.70	0.74	-2.29	0.022	0.18 (0.04–0.78)

### Ultrasound radiomics model establishment and verification

**Fig. 5:**
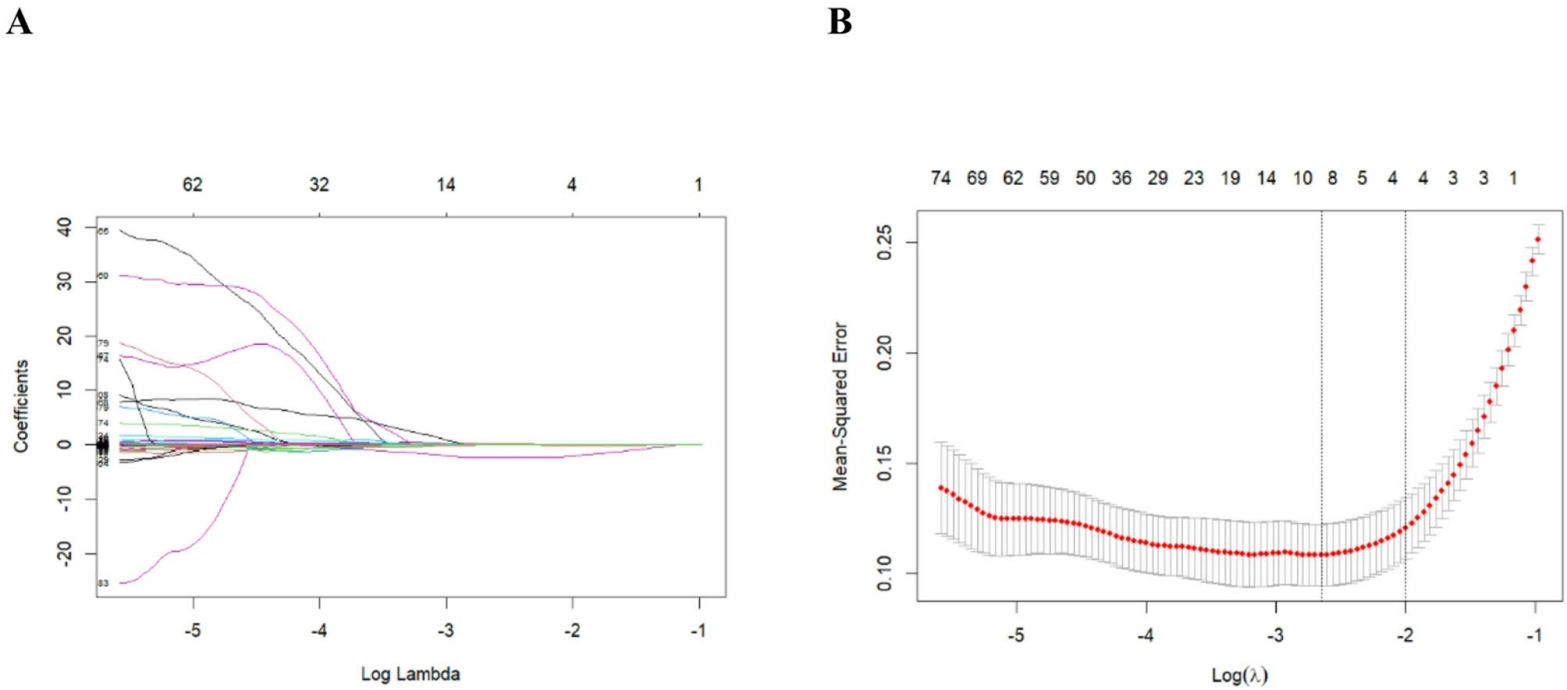
LASSO regression is used to adjust parameter selection in the training set. (**A**) For the feature with non-zero selection coefficient, the lambda value of the minimum mean square error of the training set is given; (**B**) LASSO coefficient distribution of radiomics features

**Fig. 6:**
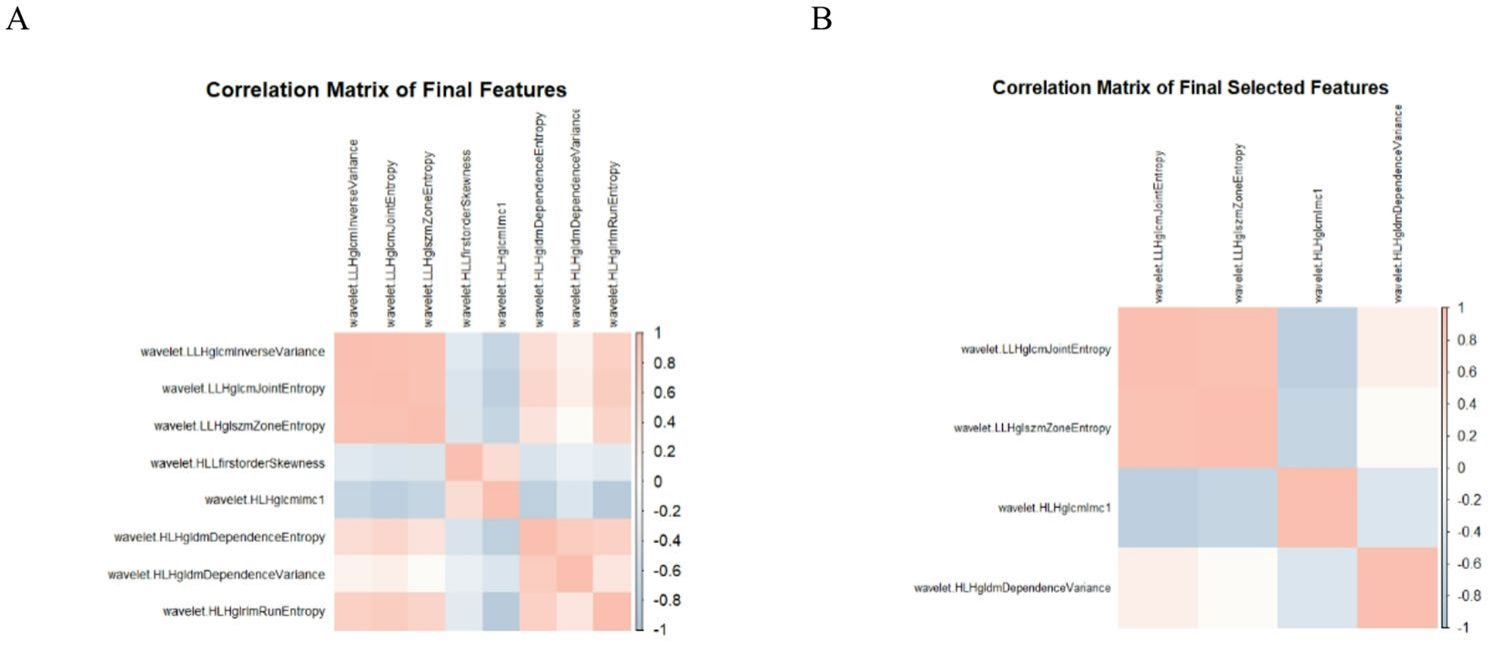
(**A**)Heatmap of Pearson correlation coefficients for selected features of LASSO regression; (**B**) Heatmap of Pearson correlation coefficients of selected features from stepwise regression. (Positive correlations are shown in red, negative correlations are shown in blue, and color shades indicate the degree of correlation)

Feature dimensionality reduction was conducted using LASSO regression. Features were filtered according to the optimal lambda value (λ = 0.07068325; Fig. 5). Four non-zero radiomic features were ultimately selected from 860 candidates: wavelet. LLH glcm Joint Entropy, wavelet. LLH glszm Zone Entropy, wavelet. HLH glcm Imc1, and wavelet. HLH gldm Dependence Variance (Table 3). Pearson correlation coefficients were calculated for the selected features, and a correlation matrix heatmap was generated (Fig. 6).

**Table 3:** Radiomic features selected using LASSO regression analysis

Variables	Estimate	Std.Error	z value	*P*
wavelet.LLH glcm Joint Entropy	-2.211	1.186	-1.864	0.062
wavelet.LLH glszm Zone Entropy	3.782	1.607	2.353	0.019
wavelet.HLH glcm Imc1	-45.800	32.338	-1.416	0.157
wavelet.HLH gldm Dependence Variance	6.009	2.839	2.116	0.034

**Fig. 7:**
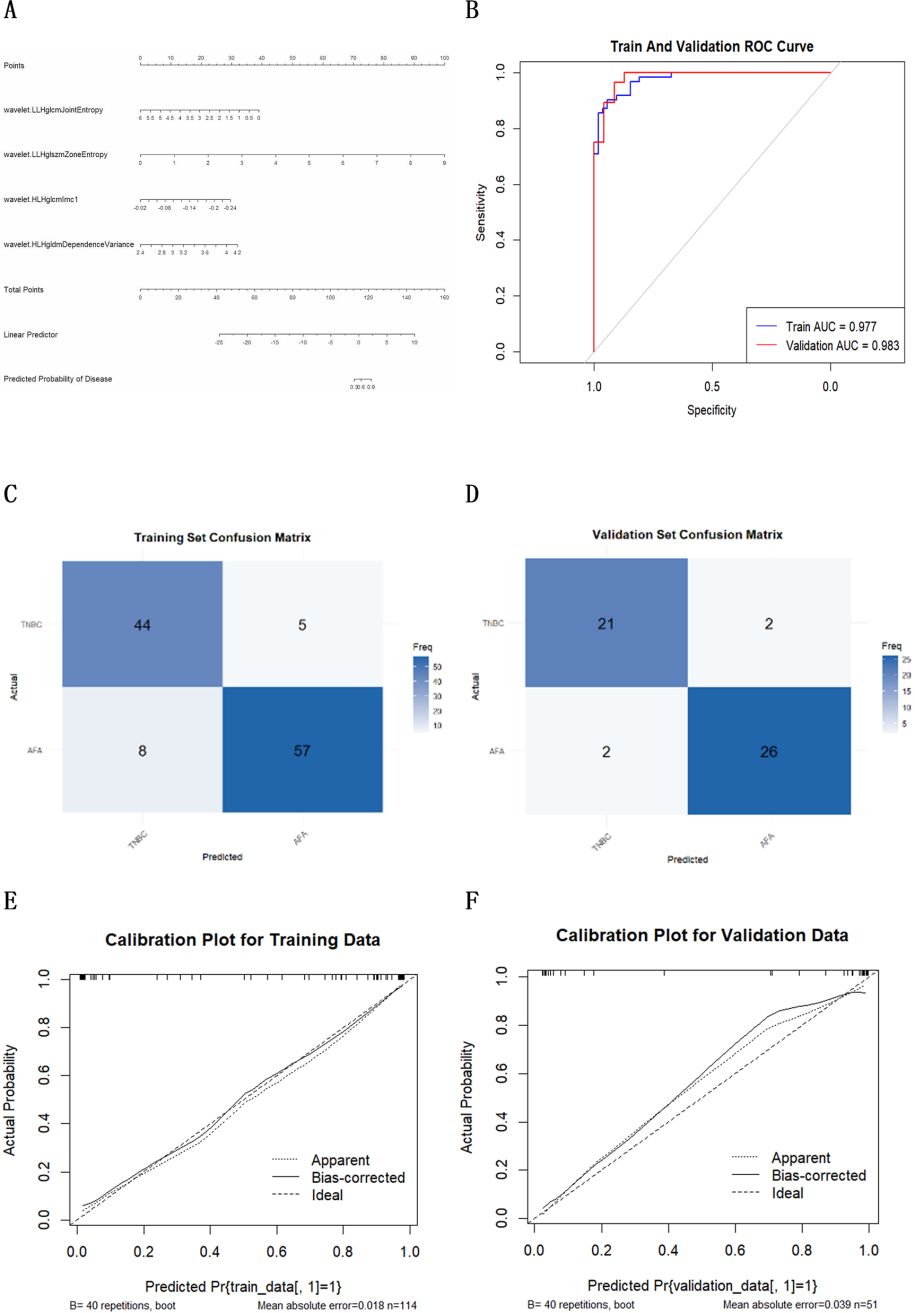
(**A**) A nomogram was developed of the final screened imaging histologic features; (**B**) ROC curves of the radiomic features model in the training cohort (blue curve) and validation cohort (red curve); (**C**, **D**) Confusion matrix plot for the training set and validation set; (**E**, **F**) The calibration curves of the radiomic features model in the training cohort and validation cohort

A nomogram for predicting TNBC was constructed based on ultrasound radiomics features (Fig. 7A). ROC curves were plotted, yielding areas under the curve (AUC) of 0.977 (95% CI: 0.957–0.977) for the training set and 0.982 (95% CI: 0.956–0.982) for the validation set (Fig. 7B). Calibration curves indicated good agreement between predicted and observed outcomes (Hosmer-Lemeshow test, *P* = 0.440). Using the optimal cut-off probability of 0.325 determined by the Youden index, confusion matrices were generated and performance metrics calculated. For the training set, the results were as follows: PPV: 0.877, NPV: 0.898 and accuracy: 0.886. For the validation set, the results were as follows: PPV: 0.929, NPV: 0.913 and accuracy: 0.922. These matrices visualised the distribution of correctly and incorrectly classified cases (Fig. 7C and D). The calibration curve was further validated using the Hosmer-Lemeshow test (*P* = 0.990), confirming high model fit (Fig. 7E and F).

## Discussion

TNBC is a highly aggressive subtype of breast cancer that lacks well-defined therapeutic targets, making accurate diagnosis essential for guiding clinical decision-making. Previous studies have demonstrated that TNBC and Atypical Fibroadenoma (AFA) share considerable imaging similarities, which complicates differential diagnosis and treatment planning [30, 31]. Consequently, reliable distinction between TNBC and AFA is of critical clinical importance. The present study evaluates the potential of ultrasound radiomics in differentiating TNBC from AFA and develops a diagnostic model that provides clinicians with a practical and valuable analytical tool [32, 33].

### Ultrasound features

In the present study, univariate analysis identified statistically significant differences in ten ultrasound features between the TNBC and AFA groups. However, multivariate regression revealed that only elasticity score, fibrous strands, and lateral shadow remained significant, underscoring their importance as key discriminators. Among these, elasticity score plays a particularly valuable role in differentiating benign from malignant lesions, as different breast tumor types exhibit varying stiffness on elastography [34]. A higher elasticity score generally indicates increased stiffness and a greater likelihood of malignancy, whereas a lower score is more consistent with fibroadenomas, aligning with findings from previous reports [35].

Fibrous strands, visualized as hyperechoic linear structures within nodules, play a critical role in breast ultrasound imaging. In this study, they emerged as an independent predictor in the conventional ultrasound model, showing a strong association with AFA (OR <1, CI approaching 0). In addition, AFA more frequently exhibited lateral acoustic shadowing than TNBC, likely reflecting its intact capsule [36]. Collectively, fibrous strands and lateral shadowing represent valuable adjunctive features for distinguishing AFA from TNBC [37].

### Ultrasound radiomics features

The selected radiomic features, including Joint Entropy and Zone Entropy, quantify specific aspects of image heterogeneity. Joint Entropy (from GLCM) reflects the complexity and randomness of the gray level co-occurrence patterns, while Zone Entropy (from GLSZM) measures the diversity in the sizes of homogeneous gray level zones. Joint Entropy (GLCM): Reflects the complexity and randomness of gray-level co-occurrence, corresponding to TNBC’s higher cellular heterogeneity and disorganized tissue architecture. Zone Entropy (GLSZM): Measures the diversity of homogeneous gray-level zones, indicating the variability of internal tissue structures in TNBC.Imc1: Quantifies pixel correlation; lower Imc1 values suggest more heterogeneous internal structures, consistent with TNBC’s aggressive biology. Dependence Variance: Captures inhomogeneity within tissue regions; higher values indicate more complex internal texture, reflecting microstructural complexity of TNBC lesions.

TNBC typically has higher cellular heterogeneity and more complex tissue structures, which is reflected in higher texture entropy values on imaging. Imc1 quantifies the correlation between image pixels. The internal structure of TNBC may be more heterogeneous, potentially resulting in lower pixel correlation on imaging. Higher dependence variance may indicate more inhomogeneous internal tissue structures in TNBC, characterized by more complex internal texture. These texture features, extracted by wavelet transformation, can capture the complexity, heterogeneity and structural differences within breast cancer lesions. TNBC’s characteristic high cellular heterogeneity and aggressive biology are often reflected in distinct radiomics features compared to other breast lesions [38, 39].

In this study, 860 radiomic features were extracted from ultrasound images using pyradiomics software. Significant multicollinearity exists between the feature parameters. Features were extracted by Spearman and LASSO algorithmic methods and bi-directional stepwise regression to minimize overfitting. As a new feature selection method, Spearman and LASSO can achieve less redundancy and more reliable radiomics features. Ultimately the four radiomic features selected in this study showed statistical differences between the two groups, indicating that these features may capture higher tissue heterogeneity or complexity in TNBC compared to AFA, with significant structural differences TNBC exhibits different gray level correlation patterns compared to AFA, which may be related to variations in cellular structure or tissue composition. The significance of these features suggests that they effectively differentiate TNBC from AFA in radiomic analysis. These features are used to quantitatively analyze subtle differences in texture complexity and gray level distribution in medical imaging, which is of considerable importance. The radiomics nomogram model achieved ROC and AUC values of 0.977 in the training sets, demonstrating high discriminatory and predictive capabilities. Performance metrics (PPV, NPV, accuracy) in the validation set slightly exceeded those in the training set, indicating that the model maintained good predictive performance on new data. Future research should further validate these features and explore their potential clinical applications to improve early diagnosis and treatment outcomes for breast cancer.

### Model comparison analysis

①Performance Comparison: The conventional ultrasound model demonstrated a high AUC(0.939), which was slightly lower than the superior performance of the radiomics nomogram (AUC = 0.977).The nomogram model outperformed the ultrasound feature data model in all performance metrics (PPV, NPV, accuracy); ②Calibration and Goodness of Fit: Both models show good fit according to the Hosmer-Lemeshow test in the training set, although both showed good fit (Hosmer-Lemeshow *P* > 0.05), the calibration curves indicated that the radiomics nomogram’s predictions aligned more closely with actual outcomes than those of the conventional ultrasound model.

The conventional ultrasound model relies on the visual assessment and interpretation of intuitive imaging characteristics, emphasizing the roles of elasticity score, fibrous strands, and lateral shadow in identifying TNBC and AFA. These features directly reflect the physical properties and growth pattern of the tumor and provide a quick and easy basis for clinical identification. However, a single ultrasound imaging feature may be affected by multiple factors and has certain limitations. In contrast, by deeply analyzing the high-dimensional features in ultrasound images and extracting important parameters such as joint entropy, regional entropy, Imc1 and dependent variance, the radiomics model not only captures the textural complexity and heterogeneity of images but also reveals the detailed differences in the microstructure of the internal breast tissue. This comprehensive analysis method based on big data significantly improves the accuracy and reliability of TNBC and AFA identification [14]. Future research can focus on improving the ultrasound feature data model to enhance its predictive performance and calibration. In addition, increasing the sample size, optimizing model parameters and exploring new radiomic features could further improve the generalizability and accuracy of the model.

The radiomics nomogram could be incorporated as a decision-support tool alongside conventional ultrasound assessment, helping clinicians identify TNBC and AFA. Integration would require standardized image acquisition protocols, automated or semi-automated ROI delineation, and user-friendly software interfaces to facilitate routine clinical use. There are several limitations to this study that warrant consideration. Firstly, the relatively small sample size may have limited the statistical power to detect subtle differences. Studies with larger cohorts are needed to provide more robust evidence and enhance the reliability of the findings. Secondly, as this investigation was conducted at a single center, the homogeneity of the study population and imaging protocols may reduce variability, yet this also restricts the generalizability of the results to broader populations with diverse demographic and clinical backgrounds. Future studies should aim to overcome these limitations through larger sample sizes, multi-center validation, and more extensive exploration of clinical utility and application [40].

## Conclusions

This study systematically analyzed the ability of ultrasound radiomics analysis in the differential diagnosis of TNBC and AFA and provided a more accurate diagnostic tool for TNBC. In the future, by expanding the sample size, optimizing the model parameters, and exploring more novel imaging histology features, further validation and refinement could enhance the model’s generalizability and accuracy.

## Data Availability

The datasets used and/or analyzed during the current study are available from the corresponding author on reasonable request.

## Abbreviations

TNBC: Triple-negative breast cancer
AFA: Atypical Fibroadenoma
CDFI: Color Doppler flow imaging
ER: Estrogen receptor
PR: Progesterone receptor
HER2: Human epidermal growth factor receptor 2
ROI: Region of Interest
LASSO: Least Absolute Shrinkage Selection Operator
AIC: Akaike Information Criterion
ROC: Receiver Operating Curve
AUC: Area Under the Curve
CI: Confidence Interval
PPV: Positive Predictive Value
NPV: Negative Predictive Value
GLCM: Gray-Level Cooccurrence Matrix
GLSZM: Gray-level Size-zone Matrix
GTDM: gray tone difference matrix
